# Antimicrobial resistance genes in pathogenic *Escherichia coli* isolated from diseased broiler chickens in Egypt and their relationship with the phenotypic resistance characteristics

**DOI:** 10.14202/vetworld.2018.1082-1088

**Published:** 2018-08-09

**Authors:** Mohamed M. Amer, Hoda M. Mekky, Aziza M. Amer, Hanaa S. Fedawy

**Affiliations:** 1 Department of Poultry Diseases, Faculty Veterinary Medicine, Cairo University, P.O. 12211, Giza, Egypt; 2 Department of Poultry Diseases, Veterinary Research Division, National Research Centre, P.O. 12622, Giza, Egypt; 3 Department of Pharmacology, Faculty Veterinary Medicine, Cairo University, P.O. 12211, Giza, Egypt

**Keywords:** antibiotic resistance genes, broiler, *Escherichia coli*, isolation

## Abstract

**Aim:**

The aim of this study was to determine the relationship between phenotypic resistance and genotypic resistance of isolated serotyped pathogenic *Escherichia coli* isolates from the clinically diseased broiler.

**Materials and Methods:**

A total of 160 samples (heart, liver, kidney, and lung) were collected from 18 to 34 days old clinically diseased broiler from 40 broiler farms (3-5 birds/farm) reared in Giza and Kaluobaia Governorates for the isolation of pathogenic *E. coli*. Various *E. coli* isolates were tested for the pathogenicity based on Congo red (CR) dye binding assay. The obtained CR-positive *E. coli* isolates were subjected to serological identification using slide agglutination test. Disc diffusion test was used to study the sensitivity pattern of *E. coli* isolates to available 12 antibiotics. Polymerase chain reaction was performed for the detection of antimicrobial resistance genes in the studied pathogenic *E. coli* isolates.

**Results:**

The results revealed that 56 samples (35 %) were positive for *E. coli*. The results of the CR assay indicates that 20 isolates of 56 (35.7%) were positive and 36 isolates (64.3%) were negative. Identified *E. coli* serotypes of CR-positive isolates were 1 (O24), 2 (O44), 2 (O55), 5 (O78), 2 (O86), 1 (124), 3 (O127), 1 (O158), and 3 untyped. Resistance rate in disc diffusion test was 85% to oxytetracycline and kanamycin; 80% to ampicillin (AMP), clindamycin, and streptomycin (S); 75% to enrofloxacin; 65% to chloramphenicol; 55% to cefotaxime and gentamicin (CN); 45% to trimethoprim+sulfamethoxazole; 35% to erythromycin (ERI); and 30% to oxacillin. All strains are multidrug-resistant (MDR). Antibacterial resistance genes *CITM, ere, aac (3)-(IV), tet(A), tet(B), dfr(A1)*, and *aad(A1)* were detected in 14 (70%), 12 (60%), 12 (60%), 8 (40%), 11 (55%), 8 (40%), and 9 (45%) of tested 20 isolates, respectively. Multidrug resistance was detected in the form of resistance to 42%-83.3% of tested 12 antibiotics. Three isolates (15%) of 20 tested isolates showed a relationship between phenotype and genotype and 17 (85%) showed irregular relation. Strains are sensitive and show resistant gene (P-G+) presented in three isolates for AMP (beta-lactam), one for ERI (Macrolide), as well as five isolates for trimethoprim (pyrimidine inhibitor). *E. coli* isolates had resistance and lacked gene (P+ G-) reported meanly in one isolate for CN (aminoglycoside), two isolates for tetracycline, four isolates for ERI, seven isolates for trimethoprim, and eight isolates for S (aminoglycoside).

**Conclusion:**

The study demonstrates that *E. coli* is still a major pathogen responsible for disease conditions in broiler. *E. coli* isolates are pathogenic and MDR. Responsible gene was detected for six antibiotics in most of the isolates, but some do not show gene expression, this may be due to few numbers of resistance genes tested or other resistance factors not included in this study.

## Introduction

*Escherichia coli* are responsible for avian colibacillosis, acute and largely systemic disease that promotes significant economic losses in poultry industry worldwide because of mortality increase, medication costs, and condemnation of carcass [1]. Several reports are available on the involvement of serotypes of *E. coli* in poultry diseases [2]. The pathogenic and non-pathogenic *E. coli* strains in poultry are differentiated based on the virulence which has been attributed to various factors including fimbriae, production of colicin, motility, and embryo lethality. Hence, detection of these strains become important for effective treatment and control [3].

Resistant to multiple antimicrobials is called multidrug-resistant (MDR) or sometimes superbugs [4]. Many suggestions about the transfer of resistance genes between bacterial generations may be inherent or acquired through vertical or horizontal transfer (transformation, conjugation, and transduction) mechanisms. The development of antibiotic resistance is usually associated with genetic changes encoded by chromosomal and plasmid genes harbored by bacterial organisms [5]. Genetic changes either to the acquisition of resistance genes or the mutations in elements relevant for the activity of the antibiotic in bacteria. It was found that, in some situations, resistance can be achieved without any genetic alteration; this is called phenotypic resistance. Non-inherited resistance is associated with specific processes such as a stationary growth phase, growth in biofilms, or persistence [6]. Phenotypic resistance is determined by the genotype [7]. Understanding of the movement of antimicrobial-resistant organisms or resistance genes into food animal life stock and further into people from the food chain and the role of the global food systems in the dissemination of resistance as well as suggested approach for antimicrobial resistance surveillance were discussed [8].

Food animals and their production environments are source and reservoirs of both resistant bacteria and resistance genes that could be transferred to humans either by direct contact with animals or indirectly through the food production chain [9,10] or as a result use of animal wastes in agriculture as a fertilizer [11,12]*. E. coli* bacteria often carry multiple drug resistance plasmids and, under stress, readily transfer those plasmids to other species. Mixing of species in the intestines allows *E. coli* to accept and transfer plasmids from one to other bacteria, and this process called horizontal gene transfer [13].

Many authors had clarified a lot about the antibiotic resistance profile of bacteria including *E. coli* in poultry farms in Egypt [14-17] but not much focused on the relationship between phenotypic resistance and genotypic resistance. Therefore, the aim of this study was to determine the relationship between phenotypic resistance and genotypic resistance of isolated serotyped pathogenic *E. coli* isolates from the clinically diseased broiler.

## Materials and Methods

### Ethical approval

This study was approved by the Institutional Animal Ethics Committee and in accordance with local laws and regulations.

### Sample collection

Samples were collected in winter from December 2016 to February 2017. A total of 160 samples (heart, liver, kidney, and lung) were collected from 18 to 34 days old sacrificed clinically diseased broiler from 40 broiler farms (3-5 birds/farm) reared in Giza and Kaluobaia Governorates, Egypt, in separate zipper-lock bag, kept in ice box, and immediately transported to the laboratory. Dead chickens showed lesions of colibacillosis or chronic respiratory disease.

### E. coli isolation

A loopful from each organ were inoculated onto the nutrient broth and incubated aerobically at 37°C for 12 h. Loopfuls from incubated nutrient broth were streaked onto Eosin methylene blue (EMB) agar plates and incubated for 24 h at 37°C. The suspected colony was picked up and streaked on the MacConkey’s agar plates and then incubated for another 24-48 h at 37°C. The suspected lactose-fermented colonies were picked up and kept in semi-solid agar for morphological and biochemical identification [18,19].

### In vitro pathogenicity testing

Various serotypes were tested for the pathogenicity based on Congo red (CR) dye binding assay according to Berkhoff and Vinal [20]. Each isolate was cultured on a separate plate of Trypticase soy agar supplemented with 0.003% CR dye (Sigma) and 0.15% bile salts. The appearance of deep brick red colonies after incubated at 37°C for 24 h was recorded as positive.

### Serological typing of E. coli

The obtained 20 CR-positive *E. coli* isolates were subjected to serological identification [21] using slide agglutination test using polyvalent and monovalent diagnostic *E. coli* antisera.

### Antibacterial susceptibility test

*E.coli* isolates were subjected to disc diffusion test to study their sensitivity pattern to available antibiotics. Twelve tested antibacterial agents and their corresponding concentrations were demonstrated as follows: Chloramphenicol 30 µg/disk (C30), gentamicin 10 µg/disk (CN), erythromycin 15 µg/disk (ERI), cefotaxime 30 µg/disk (CTX), ampicillin (AMP) 10 µg/disk (A), streptomycin 10 µg/disk (S), enrofloxacin (ENR) 5 µg/disk, oxytetracycline 30 µg/ml (T30), oxacillin 30 µg/ml (OX), kanamycin 30 µg/disk (K), trimethoprim+sulfamethoxazole 2.25/23.75 µg/disk (SXT), and clindamycin 2 µg/disk (DA). The plates were inoculated with *E. coli* suspension adjusted to 1.5×10^8^ CFU/ml corresponding to tube no. 0.5 McFarland standard. The inoculated plates were incubated aerobically at 37 C for 18-24 h, the susceptibility of the *E. coli* isolates to each antimicrobial agent was measured, and the results were interpreted according to CLSI [22]. Resistant to multiple antimicrobials was considered as MDR.

### Polymerase chain reaction (PCR)

Four colonies of each of the tested MDR *E. coli* isolates were individually subcultured overnight in Luria-Bertani broth (Merck, Germany), and genomic DNA was extracted using a Genomic DNA purification kit (Fermentas, Germany) according to the manufacturer’s instructions. PCR amplification and antimicrobial resistance genes were performed according to Peirano *et al*. [23]. The lysates were used as template in PCR to detect *CITM* in AMP-resistant isolates, *ere* in ERI-resistant isolates, *aac (3)-(IV)* in gentamicin-resistant isolates, *tet(A)* and *tet(B)* in tetracycline (TC)-resistant isolates, *dfr(A1)* in trimethoprim-resistant isolates, and *aad(A1)* in S-resistant isolates (Table-1) [24-26]. Positive controls were field samples previously confirmed positive (for the related genes) in our biotechnology laboratory, while negative controls were PCR mixture in addition to sterile distilled water.

**Table-1:** PCR primers, amplicons size, and temperature conditions used for the detection of antimicrobial resistance genes.

Antimicrobial	Resistance gene	Primers sequence	Amplicon size (bp)	Annealing temperature	References
AMP	*CITM*	F: TGGCCAGAACTGACAGGCAAA	462	47	[24]
		R: TTTCTCCTGAACGTGGCTGGC			
ERI	ere	F: GCCGGTGCTCATGAACTTGAG	419	52	[24]
		R: CGACTCTATTCGATCAGAGGC			
Gentamicin	*aac (3)*- (IV)	F: CTTCAGGATGGCAAGTTGGT	286	55	[24]
		R: TCATCTCGTTCTCCGCTCAT			
Tetracycline	*tet* (A)	F: GGTTCACTCGAACGACGTCA	577	57	[25]
		R: CTGTCCGACAAGTTGCATGA			
	*tet* (B)	F: CCTCAGCTTCTCAACGCGTG	634	56
		R: GCACCTTGCTGATGACTCTT			
Trimethoprim	*dfr* (A1)	F: GGAGTGCCAAAGGTGAACAGC	367	45	[26]
		R: GAGGCGAAGTCTTGGGTAAAAAC			
S	*aad* (A1)	F: TATCCAGCTAAGCGCGAACT	447	58	[25]
		R: ATTTGCCGACTACCTTGGTC			

PCR=Polymerase chain reaction, AMP=Ampicillin, S=Streptomycin, ERI=Erythromycin

## Results and Discussion

*E. coli* infections in birds cause many clinical manifestations which are characterized by a respiratory disease that is frequently followed by a generalized infection which ends by death [1]. Sampled chickens from diseased flocks show postmortem lesions including general congestion, with characteristic fibrinous lesions (airsacculitis, perihepatitis, and pericarditis) and fatal septicemia [1,27].

The results of *E. coli* isolation and identification from diseased field samples revealed that 56 samples (35%) were positive. Similar isolation rate was detected [16,28-30]. El Gaber and El-Gohary [31] recovered *E. coli* from 59% of 150 septicemic broilers, aged 25 days.

The results of the CR-binding assay indicate that 20 isolates of 56 (35.7%) were positive and 36 isolates (64.3%) were negative. The results of *in vitro* pathogenicity testing were in agreement with Berkhoff and Vinal [20], who reported a strong correlation between expression of CR phenotype and virulence in avian *E. coli*. Pathogenic *E. coli* can be identified by their ability to bind CR and produce brick red colonies [32,33]. A similar result of 28.6% of virulent avian *E. coli* isolates was CR positive [16]. The characteristic of CR binding constitutes a moderately stable, reproducible, and easily distinguishable phenotypic marker. Nevertheless, Yoder [34] has reported that CR binding did not correlate well with pathogenicity.

The most predominant serotypes were 1 (O24), 2 (O44), 2 (O55), 5 (O78), 2 (O86), 1 (124), 3 (O127), 1 (O158), and 3 untyped (Table-2). A wide variety of *E. coli* serotypes and non-subtypes from broiler in Egypt were also reported [14-17,35,36].

**Table-2:** Serotypes and percentages of 20 CRpositive *E. coli* isolates.

*E. coli* serotype	Number of isolate (%)
O24	1 (5)
O44	2 (10)
O55	2 (10)
O78	5 (25)
O86	2 (10)
0124	1 (5)
O127	3 (15)
O158	1 (5)
un-typed	3 (15)

*E. coli=Escherichia coli,* CR=Congo red

Isolate resistance rate was 85% to oxytetracycline and K; 80% to AMP, DA, and S; 75% to ENR; 65% to C; 55% to CTX and CN; 45% to SXT; 35% to ERI; and 30% to OX (Table-3). Isolates were resistant to 5-10 tested drugs. This result showed that all isolated strains are MDR (Tables-3 and 4). Similar results were reported by Guerra *et al*. [37]. High resistant to TC and S [38,39], AMP [40-42], and cephalexin [43] was recorded. Lower resistance rate (25.3%) was recorded by Ozawa *et al*.[42].

**Table-3:** Antimicrobial sensitivity pattern of tested *E. coli* isolates (n=20).

Isolate No.	Antibacterial agents												Drugs resistant
	
	C30	CN	CTX	A	S	ERI	ENR	T30	OX	K	SXT	DA	n (%)
1	S	R	S	R	R	R	R	R	S	R	R	R	9 (75)
2	R	S	S	S	R	R	R	R	I	R	S	R	7 (58)
3	I	R	R	S	I	R	R	R	R	R	R	I	8 (67)
4	R	R	S	R	R	I	R	R	S	I	S	R	7 (58)
5	I	I	S	S	R	R	I	R	R	R	R	S	6 (50)
6	R	R	S	R	R	R	R	R	S	R	R	R	10 (83.3)
7	R	R	S	R	R	R	R	R	R	R	S	R	10 (83.3)
8	R	R	R	S	R	R	R	R	S	R	R	R	10 (83.3)
9	I	I	S	R	R	R	R	R	S	R	R	I	7 (58)
10	R	S	S	S	I	R	R	R	I	R	S	R	6 (50)
11	I	S	S	R	R	S	I	S	R	R	R	S	5 (42)
12	R	R	R	S	R	R	R	R	S	R	R	R	10 (83.3)
13	R	S	S	R	R	R	R	R	I	R	I	R	8 (67)
14	R	R	R	S	R	S	R	I	S	S	S	R	6 (50)
15	S	S	S	R	R	S	R	S	R	R	S	R	6 (50)
16	R	S	R	S	S	R	I	R	S	R	S	R	6 (50)
17	I	R	S	R	R	R	S	R	R	R	R	R	9 (75)
18	R	R	R	R	S	R	R	R	S	S	S	R	8 (67)
19	R	S	R	S	R	R	I	R	S	R	S	R	7 (58.3)
20	R	R	S	R	R	R	R	R	I	R	I	R	9 (75)
Total R	13	11	7	11	16	16	15	17	6	17	9	16	
%	65	55	35	55	80	80	75	85	30	85	45	80	

R=Resistant, S=Sensitive, I=Intermediate, C30=Chloramphenicol 30 μg/disk, CN=Gentamicin 10 μg/disk, ERI=Erythromycin 15 μg/disk, CTX=Cefotaxime 30 μg/disk, A=Ampicillin 10 μg/disk, S=Streptomycin 10 μg/disk, ENR=Enrofloxacin 5 μg/disk, T30=Oxytetracycline 30 μg/ml, OX=Oxacillin 30 μg/ml, K=Kanamycin 30 μg/disk, SXT=Trimethoprim+sulfamethoxazole 2.25/23.75 μg/disk, DA: Clindamycin 2 μg/disk, *E. coli=Escherichia coli*

Multidrug resistance was detected in the form of resistance to 5 (42%)-10 (83.3%) of tested 12 antibiotics (Table-3). Similar results were reported [40,44,45]. MDR *E. coli* and other *Enterobacteriaceae* isolates are characterized by non-susceptibility to at least one agent in three or more antibiotic categories [45,46].

Phenotypic resistances were verified by PCR amplification and could be traced back to the genes. Of the 20 isolates tested for the presence of antibacterial resistance genes, 14 (70%) were positive to *CITM*, 12 (60%) for *ere* and *aac (3)-(IV)* genes, 8 (40%) for *tet(A)*, 11 (55%) for *tet(B)*, 8 (40%) for *dfr(A1)*, and 9 (45%) for *aad(A1)*. The obtained results of antimicrobial resistance genes agree with Guerra *et al*. [37], and Szmolka *et al*. [47]. Van *et al*. [24] detected *tetA* and *tetB* genes in *E. coli* isolated from broiler chickens. Ying *et al*. [44] found that 97% of the AMP-resistant mechanism could be explained by the resistance gene *TEM*; 90% of the TC was explained by *tet(A)*, *tet(B)*, and *tet(M)* resistance genes; and 96% of the SXT resistances could be explained by *sul(I)*, *sul(II)*, and *sul(III)* resistance genes.

Multiresistance genes >2 were detected in 17 isolates (85%), of them 12 isolates (60%) showed 4-7 resistance genes (Table-4). *E. coli* had been reported as a contributor to disseminate antibiotic resistance genes in natural environments [48,49]. One TC gene, either *tet(A)* or *tet(B)*, was detected in 75% of isolates as reported by Henriques *et al*. [50] whom screening of *tet* genes by PCR showed that 88% of the isolates carried at least one of the six tested genes. Three isolates (15%) of 20 tested isolates showed a relationship between phenotype and genotype, while 17 (85%) showed irregular relation (Table-5). This result is agreed with those reported in *E. coli* and Salmonella isolated from calves showed similar antimicrobial drug resistance patterns and several differences in resistance gene profile [51], and phenotype-genotype mapping is suggested to be complex and includes various mutations that cause similar phenotypic changes in analyses of phenotypic and genotypic changes of antibiotic-resistant *E. coli* strains [52].

**Table-4:** Phenotype antibiotic resistance of *E. coli* in relation to detected genotype.

Isolate No	Phenotype							Genotype							
	
	A	ERI	CN	T30	SXT	S	Number of antibiotic resistant	*CITM*	*ere*	*aac (3)*(IV)	*tet (A)*	*tet (B)*	*dfr (A1)*	*aad (A1)*	Number of gens
1	R	R	R	R	R	R	6	+	+	+	+	-	+	-	5
2	S	R	S	R	S	R	3	-	-	-	+	+	+	+	4
3	S	R	R	R	R	I	4	-	-	+	-	+	-	-	2
4	R	I	R	R	S	R	4	+	-	+	-	+	+	+	5
5	S	R	I	R	R	R	4	+	+	+	-	+	-	-	4
6	R	R	R	R	R	R	6	+	+	+	-	+	-	-	4
7	R	R	R	R	S	R	5	+	+	-	-	+	-	-	3
8	S	R	R	R	R	R	5	-	+	+	-	+	-	-	3
9	R	R	I	R	R	R	5	+	+	+	+	-	-	-	4
10	S	R	S	R	S	I	2	-	-	-	+	-	-	+	2
11	R	S	S	S	R	R	3	+	-	-	-	-	+	+	3
12	S	R	R	R	R	R	5	-	+	+	-	+	-	+	4
13	R	R	S	R	I	R	4	+	+	-	+	-	-	-	3
14	S	S	R	I	S	R	2	-	+	+	-	+	-	+	4
15	R	S	S	S	S	R	2	+	-	-	-	+	+	+	4
16	S	R	S	R	S	S	2	+	+	-	-	-	+	-	3
17	R	R	R	R	R	R	6	+	-	+	-	-	-	-	2
18	R	R	R	R	S	S	4	+	+	+	+	-	-	-	4
19	S	R	S	R	S	R	3	+	-	-	+	-	+	+	4
20	R	R	R	R	I	R	5	+	+	+	+	+	+	+	7
Number of positive	11	16	11	17	9	16		14	12	12	8	11	8	9	
%	55	80	55	85	45	80		70	60	60	40	55	40	45	

R = Resistant, S = Sensitive, I = Intermediate, A = Ampicillin 10 μg/disk, ERI = Erythromycin 15 μg/disk, CN = Gentamicin 10 μg/disk, T30 = Oxytetracycline 30 μg/ml, SXT = Trimethoprim + sulfamethoxazole 2.25/23.75 μg/disk, S = Streptomycin 10 μg/disk, *CITM* = Ampicillinresistant gen, *Ere* = Erythromycinresistant gene, *aac (3)(IV)*=Gentamicinresistant gene, *tet (A)* and *tet (B)*=Tetracyclineresistant genes, *dfr (A1)*=Trimethoprimresistant gene, *aad (A1)*=Streptomycinresistant gene, *E. coli = Escherichia coli*

*E. coli* isolates sensitive and has the resistant gene (P− G+) presented in three isolates for AMP (beta-lactam), one for both ERI (macrolide), as well as five isolates for trimethoprim (pyrimidine inhibitor) (Table-5). These strains appeared to harbor pseudogenes, which are defined as inactive but stable components of the genome derived by the mutation of an ancestral active gene [53] and likely to be an underestimate of the true prevalence of pseudogenes in the tested *E. coli* populations. This represents a serious limitation of an assay dependent on the detection of phenotype-genotype discrepancies with the intent to discover pseudogenes [54,55]*. E. coli* isolates had resistance and lacked gene (P+ G−) reported meanly in one isolate for CN (aminoglycoside), two isolates for TC, four isolates for ERI (macrolide), seven isolates for trimethoprim (pyrimidine inhibitor), and eight isolates for S (aminoglycoside) (Table-5). These results can be attributed to mutations in the promoter and attenuator sequences of chromosomal genes as reported by Daniels *et al.* [56], Mulvey *et al*. [57], and Escudero *et al*. [58]. All isolates phenotypically resistant to OX did not have the *mecA* gene [59], and only six of seven phenotypically completely resistant to OX CNS species expressed the *mecA* gene [60].

**Table-5:** *E. coli* showing an antibiotic pattern and gene detection*P+*G− (Yellow), *P−*G+ (blue), and*P+*G+ (light green).

Isolate No	Ampicillin		Gentamycin		ERI		Tetracycline			Trimethoprim		S	

	A	*CITM*	CN	*aac (3)(IV)*	ERI	*ere*	T30	*tet (A)*	*tet (B)*	SXT	*dfr (A1)*	S	*aad (A1)*
1												R	-
2					R	-				S	**+**		
3					R	-				R	-		
4										S	**+**		
5	S	**+**								R	-	R	-
6										R	-	R	-
7			R									R	-
8										R	-	R	-
9										R	-	R	-
10					R	-							
12										R	-	R	-
13													
14					S	**+**							
15										S	**+**		
16	S	+					R	-	-	S	**+**		
17							R	-	-	R	-	R	-
19	S	**+**			R	-				S	**+**		

R=Resistant, S=Sensitive, I=Intermediate, A=Ampicillin 10 µg/disk, ERI=Erythromycin 15 µg/disk, CN=Gentamicin 10 µg/disk, T30=Oxytetracycline 30 µg/ml, SXT=Trimethoprim+sulfamethoxazole2.25/23.75 µg/disk, S=Streptomycin 10 µg/disk. *CITM*=Ampicillin-resistant gene, *ere*=Erythromycin-resistant gene, *aac (3)-(IV)*=Gentamicin-resistant gene, *tet(A)* and *tet(B)*=Tetracycline-resistant genes, *dfr(A1)*=Trimethoprim-resistant gene, *aad(A1)*=Streptomycin-resistant gene, *E. coli=Escherichia coli*

## Conclusion

The present study demonstrates the occurrence of pathogenic MDR *E. coli* in broiler chickens in Egypt. Antibiotics resistance genes were detected in most of the isolates, but some do not show gene expression, this may be due to testing for few numbers of resistance genes or other resistance factors not included in this study.

## Authors’ Contributions

MMA designed, planned this study, drafted, and revised the manuscript. HMM, AMA, and HSF shared in samples collection, performing the tests, manuscript writing, and data analysis. All authors read and approved the final manuscript.
